# Two new species of Andean gymnophthalmid lizards of the genus *Euspondylus* (Reptilia, Squamata) from central and southern Peru

**DOI:** 10.3897/zookeys.109.1304

**Published:** 2011-06-20

**Authors:** Germán Chávez, Karen Siu-Ting, Vilma Duran, Pablo J Venegas

**Affiliations:** 1 División de Herpetología, Centro de Ornitología y Biodiversidad (CORBIDI), Santa Rita N°105 Of. 202, Urb. Huertos de San Antonio, Surco, Lima, Perú; 2 Dpto. de Herpetología, Museo de Historia Natural, Universidad Nacional Mayor de San Marcos, Lima, Perú

**Keywords:** *Euspondylus*, Gymnophthalmidae, Peru, new species

## Abstract

Two new species of lizards assigned to the genus *Euspondylus* from the montane forests of the Peruvian Andes in the Pasco Department (central Peru) and Ayacucho Department (southern Peru) both at elevations of 2550 and 3450 m, respectively, are described. The new species are distinguishable from all other Peruvian and Ecuadorian species of *Euspondylus* by a unique combination of morphometric, scalation and color pattern characteristics. Natural history data for the new species and for *Euspondylus spinalis* are also provided.

## Abstract (es)

Se describe dos especies nuevas de lagartijas asignadas al género *Euspondylus* de los bosques montanos de los Andes de Perú: una del Departamento de Pasco (centro de Perú) y otra del Departamento de Ayacucho (sur de Perú), ambas a 2550 y 3450 m de altitud, respectivamente. Las nuevas especies se distinguen de las otras especies peruanas y ecuatorianas de *Euspondylus* por una combinación única de características morfométricas, escamación y patrón de coloración. Además, se presenta datos de historia natural para las nuevas especies y *Euspondylus spinalis*.

## Introduction

The family Gymnophthalmidae comprises about 36 genera and 160 species of small lizards with elongated thin bodies and relatively short limbs, which are reduced in various degrees in some species and nearly absent in others (Pianka and Vitt 2003). These New World lizards are primarily limited to tropical latitudes, but gymnophthalmid diversity is high in both the lowland Amazonian forest and foothills and the valleys and hillsides of the Andes (Presch 1980). Some species in the genera *Euspondylus*, *Opipeuter*, *Pholidobolus*, *Petracola*, *Proctoporus* and *Riama* even reach high elevation in the Andes, such as *Proctoporus bolivianus* that can be found at 4080 m elevation (Doan and Castoe 2003) in the Peruvian Andes.

Eleven species are currently assigned to *Euspondylus*. These small secretive lizards are distributed along the Andes and Tepuis between Venezuela and southeastern Peru (Mijares-Urrutia et al. 2001; Köhler 2003; Köhler and Lehr 2004). The highest diversity of the genus *Euspondylus* occurs in Peru with eight species: *Euspondylus caideni* Köhler, *Euspondylus guentheri* (O'Shaughnessy), *Euspondylus josyi* Köhler, *Euspondylus maculatus* (Tschudi), *Euspondylus nellycarrillae* Köhler and Lehr, *Euspondylus rahmi* (De Grijs), *Euspondylus simonsii* (Boulenger), and *Euspondylus spinalis* (Boulenger), all of these with distributions restricted to a few localities with elevations ranging from 800–3310 m (Mijares-Urrutia et al. 2001; Köhler 2003; Köhler and Lehr 2004).

The taxonomy of *Euspondylus* is problematic due to the unclear generic diagnosis and species assignations with members of the genus *Proctoporus*. According to the traditional generic diagnosis by Peters and Donoso-Barros (1970), *Euspondylus* is characterized by the presence of anterior nasal scales separated by rostral and frontonasal scales, and prefrontal and dorsal scales that are not granular, whereas *Proctoporus* lacks prefrontal scales and has either keeled or striated dorsal scales. Kizirian (1996) reviewed the species then referred to *Proctoporus* from Ecuador (all of which were later assigned to a resurrected genus *Riama* by Doan and Castoe 2005) and corroborated the separation of both genera by the presence (in *Euspondylus*) and absence (in *Proctoporus*) of prefrontal scales. Köhler and Lehr (2004) recognized much variation in the presence of prefrontal scales in *Euspondylus spinalis* from Peru, and suggested that the presence versus absence of prefrontal scales is not a determining character for the separation of *Proctoporus* and *Euspondylus*, questioning the separation of the two genera. Doan and Castoe (2005) separated two genera from *Proctoporus*: *Riama* and *Petracola*; however, they never addressed the taxonomic distinction between the three genera and *Euspondylus*. Therefore, until a new and well supported classification of the Andean gymnophthalmids is proposed, we follow the generic diagnosis by Peters and Donoso-Barros (1970) and Doan and Castoe (2005) to distinguish between *Euspondylus*, *Proctoporus*, *Petracola* and *Riama*.

Recent herpetological surveys in the central and southern Andes of Peru resulted in the discovery of two new species of gymnophthalmids, which are described and tentatively assigned to the genus *Euspondylus*.

## Materials and methods

The format for the description of the new species generally follows that of Köhler and Lehr (2004). For the comparisons only Ecuadorian, Peruvian, and Bolivian species of *Euspondylus*, *Opipeuter*, *Petracola*, *Proctoporus* and *Riama* were included because our purpose was to distinguish the two new species from any that could occur in sympatry or be similar. Nomenclature of scale characters follows that of Köhler and Lehr (2004). Scale sizes were measured using precision calipers and were rounded to the nearest 0.1 mm. For characters recorded on both sides, the condition on the right side is presented first. Everted hemipenes were fixed with formalin 10%. Abbreviations for museum collections are as follows: CORBIDI (Centro de Ornitología y Biodiversidad) and MUSM (Museo de Historia Natural Universidad Nacional Mayor de San Marcos, Lima, Peru) and GPS coordinates were taken using the geodetic datum WGS84.

## Results

### Euspondylus chasqui sp. n.

urn:lsid:zoobank.org:act:D08B998E-CAAD-4648-AA13-DB7AEE1C0250

http://species-id.net/wiki/Euspondylus_chasqui

Figs 1- 2 

#### Holotype.

**Figure 1.**
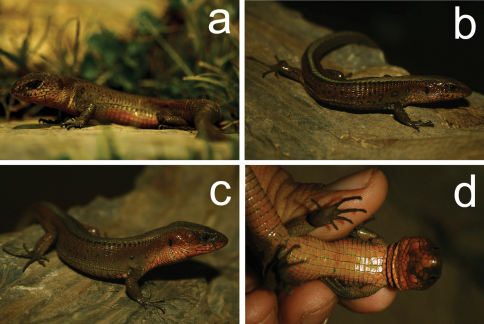
*Euspondylus chasqui*, new species, from southeastern Peru. Holotype male (CORBIDI 06963, a); female (CORBIDI 06961, b); and male (CORBIDI 06969, c, d).

(Fig. 1a) Adult male (CORBIDI 06963), Perú, Ayacucho Department, La Mar Province, Chiquintirca (13°01'59.7"S, 73°40'46.0"W), 2780 m elevation, collected by Germán Chávez on 24 August 2010.

#### Paratypes.

(Fig. 1b-d) CORBIDI 06955, 06966, 06968–69 (all adult females), 06961–62, 06965, 06967 (all adult males), 06964 (juvenile), same data as holotype; CORBIDI 08413, 08415–16, 08418–19, 08423 (all adult males), 08414, 08417, 08420–22, 08424–25, 08431–32 (all adult females), Perú, Ayacucho Department, La Mar Province, surroundings of Chiquintirca (13°01'17.4"S, 73°40'30.1"W), 2598 m elevation, collected by Vilma Duran and Karla García on 18 December 2010.

#### Diagnosis.

1) Head rounded in dorsal and lateral view, frontonasal length usually equal or slightly larger than frontal length; (2) nasoloreal suture present; (3) supraoculars four, anteriormost supraocular fused with anteriormost superciliary, all supraoculars separated from ciliaries; (4) superciliary series complete, five; (5) supralabial-subocular fusion absent; (6) postoculars three; (7) postparietals three; (8) supratympanic temporals three; (9) genials in two pairs, transverse sutures perpendicular with respect to midline of body; (10) dorsal scales rectangular, juxtaposed, keeled; (11) transverse dorsal count (enlarged rows at midbody) at midbody 20–28 in both sexes; (12) longitudinal dorsal count 35–43 in both sexes; (13) longitudinal ventral count 19–22 in both sexes; (14) lateral scale rows at midbody two or three; (15) femoral pores in males 8–11, in females 7–10; two scales between femoral pores; (16) subdigital scales on 4th finger 10–16, on 4th toe 17–26; (17) limbs overlapping, pentadactyl; digits clawed; forelimb reaching anteriorly to fourth supralabial; (18) anterior preanal plate scales paired; (19) hemipenis acapitate; flounces lacking calcified spines and forming two chevrons on distal half of hemipenis whereas basal half is covered with three transverse flounces; some asulcate flounces separated by a small expansion pleat; sulcate flounces about as wide as asulcate flounces; sulcus spermaticus single, flanked by a broad naked expansion pleat widened distally; (20) dorsum olive green, brown, or reddish brown with a middorsal pale stripe bordered by a discontinous dark line on neck and body more prominent in females than males; lateral ocelli present; ventral surfaces yellowish or reddish white; (21) transparent lower palpebral disc an undivided oval; (22) prefrontals present.

*Euspondylus chasqui* can be distinguished from other Peruvian species currently assigned to *Euspondylus* by the following character states (condition for *Euspondylus chasqui* in parentheses). *Euspondylus maculatus* and *Euspondylus guentheri*: a lower palpebral disc with vertical sections (palpebral disc an undivided oval), dorsal scales smooth or wrinkled (keeled), and longitudinal dorsal count 32–37 (35–43). *Euspondylus caideni*: by three or four superciliaries (five superciliaries), pale middorsal stripe absent (present), lateral ocelli absent (present), dorsal scales reduced in size above longitudinal band of laterals granules (not reduced), longitudinal dorsal count 41–48 (35–43). *Euspondylus josyi*: by having three supraoculars, exceptionally four, (four supraoculars), pale vertebral stripe absent (present), lateral ocelli absent (present), limb overlapping 10–13 dorsal scales (10–12), longitudinal dorsal count 29–35 (35–43) and SVL to 62.0 mm (74.0 mm). *Euspondylus rahmi*: anteriormost supraocular not fused with anterior most superciliary (fused), longitudinal dorsal count 49–54 (35–43), dorsal scales reduced in size above longitudinal band of laterals granules (not reduced) and maximum SVL 71.0 mm (74.0 mm). *Euspondylus simonsii*: a pale line between the tympanum and shoulder present (absent), dorsal scales smooth or only faintly keeled on posterior dorsum (all dorsal scales keeled), longitudinal dorsal count 33–39 (35–43) and transversal count at midbody less than 35 (40–48). *Euspondylus spinalis*: head acuminate from the dorsal and lateral view (rounded), prefrontals present or absent (prefrontals present), adpressed limbs overlapping by fewer than 10 dorsal scales rows (adpressed limbs overlapping by 10–12 dorsal scale rows), dorsal scales reduced in size above longitudinal band of laterals granules (not reduced), longitudinal dorsal count 39–46 (35–43), femoral pores on one side in females 1–6 (8–10) and small SVL, according to a population from Oso Playa, Pasco Department, ranging between 44.0–55.0 mm (SVL ranging between 50.7–74.0 mm). *Euspondylus nellycarrillae*: dorsal scales subhexagonals (rectangular), and longitudinal dorsal count 41–49 (35–43), femoral pores of one side 12–15 in males, 12–14 in females (7–10 in females, 8–11 in males), and maximum SVL = 60.0 mm (74.0 mm).

*Euspondylus chasqui* can be distinguished from all species currently assigned to *Petracola*, *Proctoporus* and *Riama* by the presence of prefrontal scales (absent in all species in these three genera). *Euspondylus chasqui* can be further distinguished by the following character states (condition for *Euspondylus chasqui* in parentheses). All Bolivian and Peruvian species of *Proctoporus* except *Proctoporus pachyurus* and *Proctoporus bolivianus*: longitudinal dorsal count fewer than 36 scale rows (35–43 scale rows). *Proctoporus pachyurus*: longitudinal dorsal count 49–59 scale rows (35–43 scale rows). *Proctoporus bolivianus*: 4–8 femoral pores in males (7–11 femoral pores). All *Petracola* and *Riama* species: lower palpebral disc with vertical sections (palpebral disc an undivided oval). Northern Ecuador species of *Riama* excluding *Riama columbiana*: no band of granular scales along the sides of body between dorsal and ventral scales (granular scales present). *Riama columbiana*: limbs not overlapping when adpressed against body in adults (limbs overlapping), superciliary series incomplete (complete), and some supraoculars in contact with ciliaries (all supraoculars separated from ciliaries).

*Euspondylus chasqui* can be distinguished from *Opipeuter xestus* (condition for *Euspondylus chasqui* in parentheses): smooth dorsal scales (keeled); a single large elongate subocular (several small subocular scales); and in hemipenis morphology, large spines at the base of the sulcus spermaticus (no such spines present in *Euspondylus chasqui*).

#### Description of the holotype.

**Figure 2.**
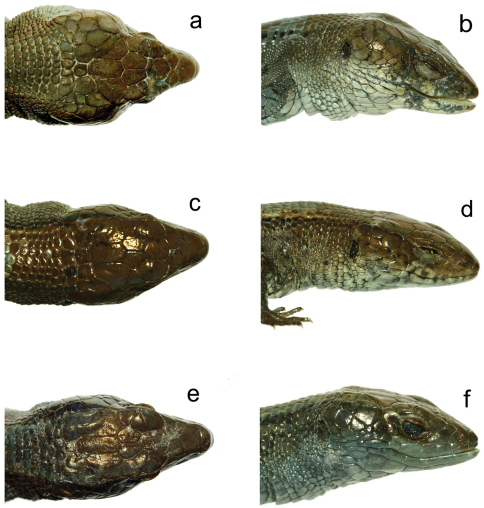
Heads of various species of *Euspondylus*. Holotype of *Euspondylus chasqui* (CORBIDI 06963, a,b), holotype of *Euspondylus oreades* (CORBIDI 07219, c,d) and *Euspondylus spinalis* (CORBIDI 07234, e,f).

Adult male (CORBIDI 06963) (Fig. 1a, 2a,b); SVL = 73.0 mm, tail (complete) length = 124.0 mm; axilla to groin distance 31.4 mm; head length 20.2 mm; head width 13.8 mm; shank length 10.7 mm.

Head scales smooth, glossy; rostral scale wider (3.2 mm) than long (1.6 mm), higher than adjacent supralabials, in contact with frontonasal, nasoloreal, and first supralabials posteriorly; frontonasal slightly longer (3.5 mm) than wide (3.4 mm), widest posteriorly, in contact with nasoloreal and frenocular laterally, prefrontals posteriorly; nasoloreal suture present; prefrontals present, in contact with each other medially, in contact with fused anteriormost superciliary-anteriormost supraocular, frontal posteriorly; frontal longer (3.7 mm) than wide (3.0 mm), anterior suture angular with point directed anteriorly, lateral sutures straight, posterior suture angular with point slightly directed posteriorly, in contact with second and third supraoculars laterally, frontoparietals posteriorly; frontoparietals pentagonal, in contact with third and fourth supraocular, parietals and interparietal posteriorly; supraoculars four, none in contact with ciliaries; superciliary series complete, anteriormost superciliary fused with anteriormost supraocular; interparietal heptagonal, longer (4.8 mm) than wide (2.7 mm), in contact with parietals laterally, postparietals posteriorly; parietals polygonal, in contact with fourth supraocular anterolaterally, temporal scales laterally, dorsalmost postocular, postparietals posteriorly; postparietals three, lateral postparietals polygonal, medial postparietal squarish; palpebral disc an undivided oval, unpigmented; frenocular squarish, in contact with nasoloreal anteriorly; postoculars three; temporals polygonal; supratympanic temporals three; supralabials seven; infralabials five; mental wider (2.9 mm) than long (1.5 mm), in contact with first infralabials, postmental posteriorly; postmental single, pentagonal, posterior suture angular, point directed posteriorly, in contact with first and second infralabials; genials in two pairs, anterior pair subquadrangular, in contact with second and third infralabials; posterior genials subpentangular, in contact with fourth and fifth infralabials laterally; scale rows between genials and collar fold (along midventral line) 12; medialmost scales of three penultimate gular scale rows slightly enlarged; posteriormost gular row enfolded posteriorly, concealing two granular scale rows; lateral neck scales rounded, smooth.

Dorsal scales rectangular, longer than wider, juxtaposed, keeled, 40 in a longitudinal count; some middorsal scales irregularly arranged; transverse dorsal count (enlarged rows at midbody) at fifth transverse ventral scale row 16, at 10th transverse ventral scale row 29, at 15th transverse ventral scale row 26; lateral scale rows at fifth transverse ventral scale row 14/ 16, at 10th transverse ventral scale row 4/4, at 15th transverse ventral scale row 3/3; lateral scales on body near insertion of forelimb small to granular; ventrals rectangular and juxtaposed; complete longitudinal ventral count 21; longitudinal ventral scale rows at midbody 12; 49 scales around midbody; anterior preanal plate scales two; posterior preanal plate scales four, all the scales at the same size; scales on tail rectangular and juxtaposed, keeled; at midventral subcaudals squarish.

Limbs pentadactyl; digits clawed; forelimb reaching anteriorly to fourth supralabial; dorsal brachial scales polygonal, of varying sizes, subimbricate, smooth; midbrachial anterodorsal scale at least twice as large as adjacent scales, smooth; anteroventral, ventral, and posteroventral scales roundish, imbricate, smooth; antebrachial scales polygonal, of various sizes; medial antebrachial scales small, rounded, smooth; dorsal manus scales polygonal, subimbricate; palmar scales small, oval, domelike; dorsal scales on fingers smooth, quadrangular, covering dorsal half of digit, overhanging supradigital scales, two on I, 5/4 on II, seven on III, nine on IV, five on V; subdigital scales 5/4 on I, 10/9 on II, thirteen on III, 14/15 on IV, 7/8 on V; anterodorsal thigh scales polygonal, at least five times as large as adjacent scales, becoming smaller ventrally, smooth; posterodorsal thigh scales small, rounded, arranged irregularly; anterior and anteromedial shank scales polygonal, subimbricate, smooth, anteriormost scales many times larger than lateral, posterolateral, and posteromedial shank scales; lateral, posterolateral, and posteromedial shank scales polygonal or roundish, juxtaposed, smooth; dorsal pes scales polygonal, subimbricate, smooth; scales on dorsal surface of digits single, quadrangular, smooth, overhanging supradigital scales, two on I, five on II, eight on III, 10/10 on IV, 6/7 on V; subdigital scales single or double, 6/7 on I, 9/10 on II, 16/15 on III, 22/21 on IV, 10/11 on V; femoral pores nine or 10; two scales between medialmost femoral pores.

The completely everted hemipenis is an acapitate organ without a medial welt; apex with two large protrusions separated by the distal end of the sulcus spermaticus; sulcus spermaticus single, flounces lacking calcified spines and forming two chevrons on distal half of hemipenis; sulcate flounces about as wide as asulcate flounces; asulcate flounces becoming shorter distally, three in the basal half and eleven in each protrusion, distal chevrons separated by a small expansion pleat; sulcus spermaticus single, flanked by a broad naked expansion pleat widened distally.

#### Coloration in preservative.

Dorsal surface of head brown, dorsal surface of body and tail bluish brown with a middorsal dark bordered pale stripe on neck and body; lateral ocelli absent; ventral surfaces dirty white suffused with pale blue.

#### Coloration in life (Fig. 1a, d).

Dorsal surface of head olive green; lateral surface of head, around the labial region yellowish orange with dark spots in each labial scale; ventral surface of head, pregular and gular region yellowish orange with dark grey spots on the genials and pregular scales. Dorsal surface of body same color as head, but with black spots in each scale around middorsal region, that form two indistinct and discontinous lines that extend from occiput to posterior hind limbs forming a dark bordered middorsal pale stripe; lateral surface of body same coloration as dorsum with one indistinct ocellus on both sides above insertion of forelimbs, some lateral scales bearing black or small orange spots; ventral surface of body reddish cream (resembling clay). Limbs similar to body, ventral surface of arms olive cream, ventral surface of legs cream. Coloration of dorsal and ventral surfaces of tail like that of body.

#### Variation(Fig. 1b-d).

In the type series, the distinctness of the pale middorsal stripe is more noticeable in females than males, whereas the lateral stripes are obscure in some. Lateral ocelli are present forming a series from three to five ocelli on each side in females, and usually one on each side in males, only one male of the type series (CORBIDI 06967) has three ocelli on each side. Sexual dimorphism is evident in the size of the femoral pores, males have bigger femoral pores than females, but not in their number (8–11 in males versus 7–10 in females), however the main differences between females and males is the SVL (maximum SVL in females = 61.0 mm, maximum SVL in males = 74.0 mm). See Table 1 for variation in selected morphometric and squamation characters in the specimens examined.

#### Etymology.

The specific epithet is based on the Quechua word “chasqui”, which refers to the messengers of the Incan empire, men who, on foot, carried the messages throughout the imperial territory in the Cordillera de los Andes where these lizards are found.

#### Distribution and natural history.

*Euspondylus chasqui* is known from two localities within a studied area of approximately 12 km² in the Río Apurímac valley (Fig 4). It inhabits secondary forests and human settlements. The individuals observed were mostly found at midday under the rocks or foraging between stones, always near medium-sized rocks that they use for hiding. The soil under these rocks is generally more damp compared to the rest of the soil around. A clutch with two eggs was found under the litter, as well as several gravid females (CORBIDI 06955, 06966, 06968–69, 08417, 08420–21, 08424, 08431–32) from the dry and wet season (August and December, 2010) containing two eggs inside the abdominal cavity. This suggests that the maximum clutch size is two and that the reproductive cycle and birth of neonates can be at least twice a year. The egg length range is 3.4–14.1 mm (*x*- = 8.3 mm, n= 20) and width range is 2.8–5.9 mm (*x*- = 4.5 mm, n=20), SVL range of gravid females is 48.5–72.7 mm. We did not see nests sharing the same area. *Euspondylus chasqui* does not occur sympatrically with any other species of *Euspondylus* or *Proctoporus*; however, a marsupial frog, *Gastrotheca rebeccae*, was found at the same location. *Euspondylus chasqui* was the most abundant species in the type locality, where 35 individuals were found in four hours by four surveyers.

**Table 1.** Morphometric and pholidosis characters in *Euspondylus chasqui* and *Euspondylus oreades*. Individuals measured for *Euspondylus chasqui* include: eleven males, thirteen females, and a juvenile; for *Euspondylus oreades*: four males, eight females, and a juvenile. Range is followed by mean value and standard deviation in parenthesis.

		*Euspondylus chasqui(n=25)*	*Euspondylus oreades(n=13)*
Max SVL (mm)	males	74.0	61.0
	females	72.7	58.0
Tail length/SVL	males	1.2–2.1 (1.6+0.3)	1.0–1.8 (1.4+0.3)
	females	1.0–2.0 (1.5+0.4)	1.0–1.9 (1.4+0.4)
Head length/Head width	males	1.0–1.8 (1.6+0.2)	1.4–1.7 (1.6+0.1)
	females	1.6–2.0 (1.9+0.1)	1.4–2.0 (1.7+0.2)
Number of femoral pores	males	8–11 (9.7+1.0)	3–8 (6.0+1.9)
	females	8–10 (9.1+0.5)	2–8 (5.8+2.3)
Number of genials		4 (4.0+0.0)	4 (4.0+0.0)
Number of postparietals		3 (3.0+0.0)	3 (3.0+0.0)
Number of supratympanic temporals		2–3 (2.6+0.5)	3 (3.0+0.0)
Number of scales around midbody		20–28 (23.2+2.4)	20–25 (22.7+1.8)
Longitudinal dorsal count		37–43 (39.1+1.7)	32–43 (39.0+2.9)
Number of longitudinal ventral scale rows		19–22 (20.1+0.9)	20–22 (21.0+0.8)
Number of transversal ventral scale rows		10–14 (12.4+0.9)	10–12 (10.6+0.9)
Lamellae under 4th finger		10–15 (13.6+1.2)	8–12 (10.1+1.1)
Lamellae under 4th toe		17–26 (21.2+1.9)	11–19 (15.2+2.0)

### Euspondylus oreades sp. n.

urn:lsid:zoobank.org:act:0C198ACA-83B5-46DF-8155-D0E31A084FBE

http://species-id.net/wiki/Euspondylus_oreades

Figs 2– 3 

#### Holotype.

**Figure 3.**
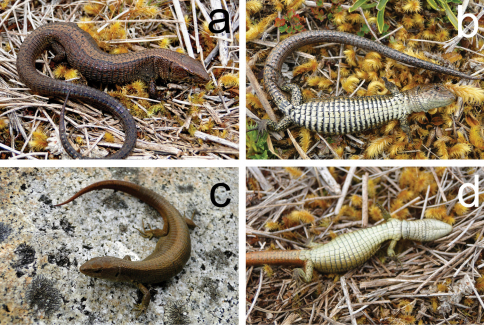
*Euspondylus oreades*, new species, from central Peru. Holotype male (CORBIDI 07219, a,b) and female (CORBIDI 07216, c,d).

(Fig 3.a-b) Adult male (CORBIDI 07219), Perú, Pasco Department, Santa Barbara (10°20'29.1"S, 75°38'27.1"W), at 3439 m of elevation, collected by Caroll Z. Landauro, Lesly Luján, Vilma Duran and Pablo J. Venegas on 23 September 2010.

#### Paratypes.

CORBIDI 07214, 07216–18, 07220, 07222, 07224–25 (all females), 07215, 07221, 07229 (all males), 07223 (juvenile), same data as holotype.

#### Diagnosis.

1) Frontonasal length usually equal or slightly larger than frontal length; (2) nasoloreal suture present; (3) supraoculars four, anteriormost supraocular fused with anteriormost superciliary, all supraoculars separated from ciliaries; (4) superciliary series complete, four; (5) supralabial-subocular fusion absent; (6) postoculars three; (7) postparietals three; (8) supratympanic temporals three; (9) genials in two pairs, transverse sutures perpendicular with respect to midline of body; (10) dorsal scales quadrangular, juxtaposed, keeled; (11) transverse dorsal count (enlarged rows at midbody) at midbody 20–26 in both sexes; (12) longitudinal dorsal count 37–43 in both sexes; (13) longitudinal ventral count 20–22 in both sexes; (14) lateral scale rows at midbody two; (15) femoral pores in males 3–8, in females 2–8; two scales between femoral pores; (16) subdigital scales on 4th finger 5–13, on 4th toe 10–19; (17) limbs overlapping, pentadactyl; digits clawed; forelimb reaching anteriorly to fourth supralabial; (18) anterior preanal plate scales paired; (19) hemipenis acapitate; flounces forming two chevrons on distal half of hemipenis whereas basal half is covered with one or two transverse flounces; asulcate flounces separated by a small expansion pleat; sulcate flounces about as wide as asulcate flounces; sulcus spermaticus single, flanked by a broad naked expansion pleat widened distally and divided by a small protrusion; (20) dorsum brown or pale brown with a middorsal pale stripe bordered by an discontinous dark line on neck and body; lateral ocelli usually absent; ventral surfaces white or creamy white; (21) transparent lower palpebral disc an undivided oval; (22) prefrontals usually present.

*Euspondylus oreades* can be distinguished from the Peruvian species of *Euspondylus* by the following character states (condition for *Euspondylus oreades* in parentheses). *Euspondylus maculatus* and *Euspondylus guentheri*: lower palpebral disc with vertical sections (palpebral disc an undivided oval), dorsal scales smooth or wrinkled (keeled), and longitudinal dorsal count 32–37 (36–43). *Euspondylus caideni*: maximum SVL = 82.0 mm (61.0 mm), pale middorsal stripe absent (present), dorsal scales reduced in size above longitudinal band of laterals granules (not reduced), longitudinal dorsal count 41–48 (36–43). *Euspondylus josyi*: pale middorsal stripe absent (present), supraoculars three, exceptionally four (always four) limb overlapping 10–13 dorsal scales (10–11) and longitudinal dorsal count 29–35 (36–43). *Euspondylus rahmi*: anteriormost supraocular not fused with anterior most superciliar (fused), dorsal scales reduced in size above longitudinal band of lateral granules (not reduced), longitudinal dorsal count 49–54 (36–43) and maximum SVL to 71.0 mm (61.0 mm). *Euspondylus simonsii*: a pale line between the tympanum and shoulder present (absent), dorsal scales smooth or only faintly keeled on posterior dorsum (all dorsal scales keeled), transversal count at midbody less than 35 scales (40–46) and longitudinal dorsal count 33–39 (36–43). *Euspondylus spinalis*: head acuminate from dorsal and lateral view (rounded), dorsal scales reduced in size above longitudinal band of lateral granules (not reduced), subdigital lamellae of fourth toe 20–24 (11–19), longitudinal dorsal count 39–46 (32–43), femoral pores on one side in females 1–6 (4–8), supracaudal scales only faintly keeled or smooth (strongly keeled) and lateral ocelli usually present (usually absent). *Euspondylus nellycarrillae*: dorsal scales subhexagonal (rectangular), longitudinal dorsal count 41–49 (36–43) femoral pores of one side 12–15 in males, 12–14 in females (6–8 in males, 2–5 in females). *Euspondylus chasqui*: superciliar series five (four), subdigital scales on the fourth toe 17–26 (13–19), femoral pores of the one side 8–11 in males, 7–10 in females (6–8 in males, 2–5 in females) and maximum SVL = 74.0 mm (61.0 mm).

*Euspondylus oreades* can be distinguished from all species currently assigned to *Petracola, Proctoporus*, and *Riama* by the presence of prefrontal scales (absent in all speciesin these three genera). *Euspondylus oreades* can be further distinguished by the following character states (condition for *Euspondylus oreades* in parentheses) from all Bolivian and Peruvian species of *Proctoporus* except *Proctoporus pachyurus* and *Proctoporus bolivianus*: longitudinal dorsal count fewer than 36 scale rows (37–43 scale rows). *Proctoporus pachyurus*: longitudinal dorsal count 49–59 (37–43). *Proctoporus bolivianus*: four or five supralabials (six or seven). All *Petracola* and *Riama* species: lower palpebral disc with vertical sections (palpebral disc an undivided oval). All northern Ecuadorian *Riama* species except *Riama columbiana*: no band of granular scales along the sides of body between dorsal and ventral scales (granular scales present). *Riama columbiana*: limbs not overlapping when adpressed against body in adults (limbs overlapping), superciliary series incomplete (complete), and some supraoculars in contact with ciliaries (all supraoculars separated from ciliaries).

*Euspondylus oreades* can be distinguished from *Opipeuter xestus* (condition for *Euspondylus oreades* in parentheses): smooth dorsal scales (keeled); having a single large elongate subocular scale (several small subocular scales); and in hemipenis morphology, large spines at the base of the sulcus spermaticus (no such spines present in *Euspondylus oreades*).

#### Description of the holotype.

Adult male (CORBIDI 07219) (Fig. 2c,d; 3a,b); SVL 55.0 mm, tail (complete) length 101.0 mm; axilla to groin distance 26.7 mm; head length 13.2 mm; head width 8.8 mm; shank length 7.2 mm. Head scales smooth, glossy; rostral scale wider (2.4 mm) than long (1.4 mm), slightly higher than adjacent supralabials, in contact with frontonasal, nasoloreal, and first supralabials posteriorly; frontonasal wider (2.8 mm) than long (2.6 mm), widest posteriorly, in contact with nasoloreal and frenocular laterally, prefrontals posteriorly; nasoloreal suture present; prefrontals present, in contact with each other medially, in contact with fused anteriormost superciliary-anteriormost supraocular, frontal posteriorly; frontal slightly longer (2.3 mm) than wide (2.2 mm), anterior suture angular with point directed anteriorly, lateral sutures straight, posterior suture angular with point directed posteriorly, in contact with second and third supraocular laterally, frontoparietals posteriorly; frontoparietals pentagonal, in contact with third and fourth supraocular, parietals and interparietal posteriorly; supraoculars four, none in contact with ciliaries; superciliary series complete, anteriormost superciliary fused with anteriormost supraocular; interparietal pentagonal, longer (3.2 mm) than wide (1.7 mm), in contact with parietals laterally, postparietals posteriorly; parietals pentagonal, in contact with fourth supraocular anterolaterally, temporal scales laterally, dorsalmost postocular, postparietals posteriorly; postparietals three, lateral postparietals pentagonal, medial postparietal squarish; palpebral disc an undivided oval, unpigmented; frenocular quadrangular, in contact with nasoloreal anteriorly; postoculars three; temporals polygonal; supratympanic temporals three; supralabials eight; infralabials seven; mental wider (2.3 mm) than long (1.4 mm), in contact with first infralabials, postmental posteriorly; postmental single, pentagonal, posterior suture angular, point directed posteriorly, in contact with first and second infralabials; genials in two pairs, both pairs subquadrangular, in contact with second and third infralabials; posterior genials subpentagonal, in contact with fourth and fifth infralabials laterally; scale rows between genials and collar fold (along midventral line) 10; medialmost scales of three penultimate gular scale rows slightly enlarged; posteriormost gular row enfolded posteriorly, concealing two granular scale rows; lateral neck scales rounded, smooth.

Dorsal scales quadrangular, longer than wide, juxtaposed, keeled, 42 in a longitudinal count; some middorsal scales irregularly arranged; transverse dorsal count (enlarged rows at midbody) at fifth transverse ventral scale row 8, at 10th transverse ventral scale row 11, at 15th transverse ventral scale row 11; lateral scale rows at fifth transverse ventral scale row 13/12, at 10th transverse ventral scale row two, at 15th transverse ventral scale row two; lateral scales on body near insertion of forelimb small to granular; ventrals rectangular and juxtaposed; one complete longitudinal at ventral count 22; longitudinal ventral scale rows at midbody 12; 29 scales around midbody; anterior preanal plate scales six; posterior preanal plate scales four (third one not totally developed), all the scales at the same size; scales on tail rectangular and juxtaposed, keeled; midventral subcaudals squarish.

Limbs pentadactyl; digits clawed; forelimb reaching anteriorly to fourth supralabial; dorsal brachial scales polygonal, of varying sizes, subimbricate, smooth; midbrachial anterodorsal scale at least twice as large as adjacent scales, smooth; anteroventral, ventral, and posteroventral scales roundish, imbricate, smooth; antebrachial scales polygonal, of various sizes; medial antebrachial scales small, polygonal, smooth; dorsal manus scales polygonal, subimbricate; palmar scales small, oval, domelike; dorsal scales on fingers smooth, quadrangular, covering dorsal half of digit, overhanging supradigital scales, two on I, five on II, five on III, six on IV, four on V; subdigital scales four on I, seven on II, 9/10 on III, 7/8 on IV, seven on V; anterodorsal thigh scales polygonal, at least two times as large as adjacent scales, becoming smaller ventrally, smooth; posterodorsal thigh scales small, rounded, arranged irregularly; anterior and anteromedial shank scales roundish, subimbricate, smooth, anteriormost scales many times shorter than lateral, posterolateral, and posteromedial shank scales; lateral, posterolateral, and posteromedial shank scales polygonal or roundish, juxtaposed, smooth; dorsal pes scales polygonal, subimbricate, smooth; scales on dorsal surface of digits single, quadrangular, smooth, overhanging supradigital scales, two on I, 4/5 on II, eight on III, ten on IV,seven on V; subdigital scales single or double, four on I, nine on II, 12/13 on III, seventeen on IV, nine on V; femoral pores seven or eight; two scales between medial most femoral pores.

The completely everted hemipenis is an acapitate organ without a medial welt; apex with two large protrusions separated by the distal end of the sulcus spermaticus; sulcus spermaticus single, flounces lacking calcified spines and forming two chevrons on distal half of hemipenis; sulcate flounces about as wide as asulcate flounces; asulcate flounces becoming shorter distally, two in the basal half and ten, in each protrusion, distal chevrons separated by a small expansion pleat; sulcus spermaticus single, flanked by a broad naked expansion pleat widened distally.

#### Coloration in preservative.

Dorsal surfaces of head, body, and tail brown with a middorsal dark bordered pale stripe on neck; bearing three lateral ocelli in both sides; ventral surface bluish white with black blotches in each scale, ventral surface of the hind limbs and forelimbs pale brown.

#### Coloration in life(Fig. 3a,b).

Dorsal surface of head pale brown; lateral surface of head dark brown, dark and white spots in each labial scale forming labial bars; ventral surface of head creamy white with irregular line on postmental, genials and post genials scales, pregular and gular region creamy white. Dorsal surface of body, on the middorsal region same color as head, but with black blotches in each middorsal scale bordering the middorsal region and forming a dark bordered pale stripe from the occipital region to the posterior insertion of the hind limbs; lateral surface of body dark brown with three ocelli above insertion forelimbs on both sides, some lateral granular scales white and some keeled lateral scales bearing black blotches; ventral surface of body creamy with with black blotches. Limbs similar to body, ventral surface of arms cream with dark spots, ventral surface of legs cream with black blotches. Coloration of dorsal and ventral surfaces of tail like that of body.

#### Variation.

In the type series, the distinctness of the pale middorsal stripes varies. However, at least the bordered stripes are visible in all specimens, whereas the lateral stripes are obscure in some, only CORBIDI 07216, is lacking the bordered stripe (Fig. 3c,d). Lateral ocelli are not present in most of the specimens except CORBIDI 07219 (three ocelli on both sides), 07221 and 07229 (both specimens with a row of ocelli on both sides). Sexual dimorphism is evident in the size of the femoral pores (bigger in males than in females) and in their number (2–5 in females versus 6–8 in males). See Table 1 for variation in selected morphometric and squamation characters in the specimens examined.

#### Etymology.

The specific name *oreades* refers to the Oreades, nymphs of Greek mythology. These feminine spirits lived and protected isolated mountains and caves, places that recall the type locality where this species was found.

#### Distribution and natural history.

**Figure 4.**
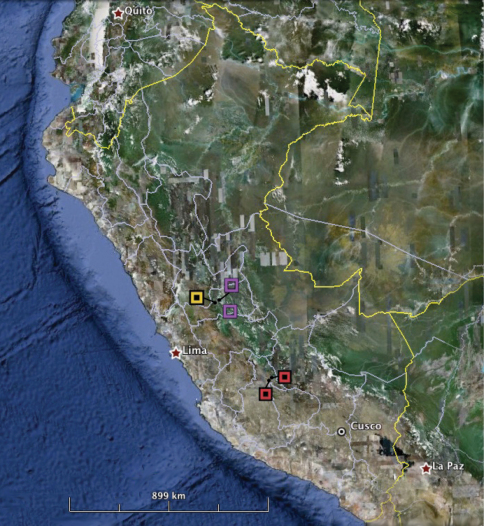
Map showing distributions of the new *Euspondylus* species. The filled-in squares represent records for both new species. Red filled-in squares correspond to *Euspondylus chasqui* and yellow filled-in square corresponds to *Euspondylus oreades*. Purple empty squares correspond to *Euspondylus spinalis*.

*Euspondylus oreades* is known only from the type locality, an isolated hill at an elevation of 3400 m in the Cordillera Oriental in central Peru, inside Yanachaga-Chemillen National Park (Oxapampa Bioesphere Reserve) (Fig. 4). Individuals were found in grassland (Puna habitat) under rocks, fallen trunks, moss, and under the base of terrestrial spiny bromeliad (*Puya* sp.) by the day. Only the marsupial frog *Gastrotheca griswoldi* was found sympatric with *Euspondylus oreades*. A total of 33 individuals of *Euspondylus oreades* were found in seven hours of survey by four herpetologists. Four nests of the new species of *Euspondylus* were found under the rocks and the number of eggs found per nest vary from two to 15. Two eggs of *Euspondylus oreades* hatched during the surveys and the new hatchlings ran to hide immediately after leaving the egg shell. One of these hatchlings was collected (CORBIDI 07223) and measured (SVL = 23.0 mm). Six females were collected, five of them contained eggs in their oviducts, only CORBIDI 07216 contained one egg, the rest of mature females contained two eggs, SVL range of these specimens was 53.0–61.0 mm. Egg length ranged from 3.2–13.2 mm (*x*- = 8.6 mm, n= 9) and width ranged from 3.6–5.7 mm (*x*-= 3.7 mm, n=9). The ornithological team in Santa Barbara collected one Variable Hawk *Buteo polyosoma* (CORBIDI/FHC 245) that contained three whole *Euspondylus oreades* individuals in the crop and the remains of three other individuals in the stomach. Furthermore, the team collected one Andean Caracara *Phalcoboenus megalopterus* (CORBIDI/WV 315) with remains of an unassigned *Euspondylus* species in its stomach. These findings could suggest that these lizards can be found in the open, but for *Euspondylus oreades* we did not have such observations. However, one of us (GC) found *Euspondylus chasqui* running between stones when sampling, which might be a behavior that could occur in *Euspondylus oreades* as well.

#### Remarks.

Most of the *Euspondylus* species occur on, isolated points in the Andes (Kizirian 1996; Köhler 2003; Köhler and Lehr 2004), in montane forests or transition areas between montane forests and grasslands. Several genera from the families Gymnophthalmidae and Iguanidae have been recorded in the high Andes (Fritts 1972; Cadle 1991; Laurent 1992; Laurent 1998). Of these *Anadia*, *Euspondylus, Petracola*, *Pholidobolus*, *Proctoporus* and *Riama* (Gymnophthalmidae) and *Stenocercus* and *Liolaemus* (Iguanidae) are the only genera found above 3000 meters of elevation.

Both species described in this paper, *Euspondylus chasqui* and *Euspondylus oreades*, are abundant at their respective type localities and, where surveyed, are not sympatric with any other lizard species. The only species that is distributed close to *Euspondylus oreades* is *Euspondylus spinalis* (Fig. 2 e,f). Even though there is no natural history data published for *Euspondylus spinalis*, we have observed that the latter occurs in montane habitats, while *Euspondylus oreades* occurs in grasslands. We have also found that *Euspondylus spinalis*, was the most abundant species at two localities: Chacos Community (0°35'24.2"S, 75°16'24.4"W, 1986 m) and Oso Playa Road (10°19'21.5"S, 75°35'03.1"W, 2000 m), in the Pasco Department (Fig. 4), with 14 individuals found in two hours by one surveyer and 87 individuals in two hours by two surveyers, respectively. Only in Chacos did we find *Euspondylus spinalis* in sympatry with the iguanid lizard *Stenocercus boettgeri*, even though, *Stenocercus boettgeri* was not abundant (only three individuals recorded). Given the high abundances observed of these gymnophthalmid lizards, it is likely that they play an important role in the lizard community composition, and apparently, in the trophic chain of certain major predators, as evidenced by the records of *Euspondylus* lizards found in the stomach contents of *Buteo polyosoma* and *Phalcoboenus megalopterus*.

## Supplementary Material

XML Treatment for Euspondylus chasqui

XML Treatment for Euspondylus oreades
